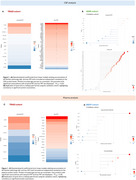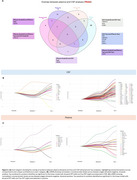# Inflammation‐related proteomic changes in response to amyloid and tau pathologies

**DOI:** 10.1002/alz70856_106582

**Published:** 2026-01-08

**Authors:** Ilaria Pola, Nicholas J. Ashton, Marco Antônio De Bastiani, Luiza Santos Machado, Guilherme Povala, Wagner S. Brum, Nesrine Rahmouni, Kübra Tan, Marisa N. Denkinger, Stijn Servaes, Joseph Therriault, Tharick A Pascoal, Kaj Blennow, Henrik Zetterberg, Eduardo R. Zimmer, Pedro Rosa‐Neto, Andrea L. Benedet

**Affiliations:** ^1^ Department of Psychiatry and Neurochemistry, Institute of Neuroscience and Physiology, The Sahlgrenska Academy, University of Gothenburg, Mölndal, Sweden; ^2^ Centre for Age‐Related Medicine, Stavanger University Hospital, Stavanger, Norway; ^3^ Banner Alzheimer's Institute, Phoenix, AZ, USA; ^4^ King's College London, London, United Kingdom; ^5^ Department of Psychiatry and Neurochemistry, Institute of Neuroscience and Physiology, University of Gothenburg, Mölndal, Gothenburg, Sweden; ^6^ NIHR Biomedical Research Centre for Mental Health and Biomedical Research Unit for Dementia at South London and Maudsley, NHS Foundation, London, United Kingdom; ^7^ Universidade Federal do Rio Grande do Sul, Porto Alegre, RS, Brazil; ^8^ Department of Psychiatry and Neurochemistry, Institute of Neuroscience and Physiology, The Sahlgrenska Academy, University of Gothenburg, Gothenburg, VG, Sweden; ^9^ University of Pittsburgh, Pittsburgh, PA, USA; ^10^ Universidade Federal do Rio Grande do Sul, Porto Alegre, Rio Grande do Sul, Brazil; ^11^ McGill University, Montreal, QC, Canada; ^12^ Montreal Neurological Institute, Montreal, QC, Canada; ^13^ Banner Sun Health Research Institute, Sun City, AZ, USA; ^14^ Institute of Neuroscienace and Physiology, University of Gothenburg, Mölndal, Västra Götaland, Sweden; ^15^ UK Dementia Research Institute, UCL Institute of Neurology, University College London, London, England, United Kingdom; ^16^ Department of Neurodegenerative Disease, UCL Queen Square Institute of Neurology, University College London, London, ‐, United Kingdom; ^17^ Clinical Neurochemistry Laboratory, Sahlgrenska University Hospital, Mölndal, Västra Götalands län, Sweden; ^18^ UK Dementia Research Institute, University College London, London, United Kingdom; ^19^ Hong Kong Center for Neurodegenerative Diseases, Hong Kong, Hong Kong, China; ^20^ University of Wisconsin School of Medicine and Public Health, Madison, WI, USA; ^21^ UFRGS, Porto Alegre, Brazil; ^22^ Brain Institute of Rio Grande do Sul ‐ Pontifícia Universidade Católica do Rio Grande do Sul, Porto Alegre, Rio Grande do Sul, Brazil; ^23^ Department of Neurology and Neurosurgery, McGill University, Montréal, QC, Canada

## Abstract

**Background:**

Emerging evidence underscores the importance of neuroinflammation in the progression of Alzheimer's disease (AD) pathophysiology. Recent studies indicate the involvement of inflammatory mechanisms in amyloid‐β (Aβ) and tau deposition in the brain. Due to the complexity of the immune responses and the intricate interplay between the peripheral and the central nervous systems, identifying biomarkers that reflect the brain inflammatory changes in AD has been challenging. With this study, we sought to characterize immune‐related proteins in cerebrospinal fluid (CSF) and plasma, in relation to Aβ and tau PET.

**Methods:**

Participants from the Translational Biomarker for Aging and Dementia Cohort (TRIAD) incorporating within the AD spectrum, and with available Aβ PET ([^18^F]AZD4694) and tau PET ([^18^F]MK6240), had plasma (*n* =  376) and CSF (*n* =  277) samples analyzed with the NULISA technology (Alamar Biosciences®). A total of 309 inflammation‐related proteins were selected and included in our analysis. Validation cohorts were used to generalize the results: ADNI had CSF samples (*n* = 384) analyzed with the SomaScan technology (SomaLogic^®^); and BBDP had plasma samples (*n* = 253) analyzed with NULISA technology (Alamar Biosciences^®^). Standardized (Std) β coefficients from linear models relating Aβ‐ and tau‐PET SUVR (both included as independent variables) to the CSF and plasma protein levels. Models included age and sex and as covariates.

**Results:**

Several proteins exhibit distinct patterns in relation to Aβ and tau pathologies. Notably, specific proteins show strong positive associations with global Aβ‐PET levels, independent of tau‐PET levels, while others are positively associated with global tau‐PET levels, independent of Aβ‐PET levels (Figure 1). Validation in ADNI (plasma data) and BBDP cohort (CSF data) confirmed these findings. A Venn diagram illustrates unique and shared significant proteins across different groups and matrices (Figure 2a). Furthermore, LOESS curves revealed that specific proteins increase or decrease along the disease pseudo‐time, highlighting their potential roles in disease progression (Figure 2b, 2c).

**Conclusion:**

Overall, using a multi‐omics approach, this preliminary analysis provided new insights on key proteins and molecular inflammatory pathways that co‐occur with, and follow the accumulation of, Aβ and tau load along the AD continuum. This analysis will be further expanded and detailed in more cohorts.